# Ist eine Delegation ärztlicher Leistungen auf rheumatologische Fachassistenten bei der Evaluierung von Patienten mit Verdacht auf ankylosierende Spondylitis möglich? – Ergebnisse der PredAS-Studie

**DOI:** 10.1007/s00393-020-00838-8

**Published:** 2020-07-21

**Authors:** U. Kiltz, I. Spiller, J. Sieper, J. Braun

**Affiliations:** 1 Rheumazentrum Ruhrgebiet und Ruhr-Universität Bochum, Claudiusstr 45, 44649 Herne, Deutschland; 2 Medizinische Klinik für Gastroenterologie, Infektiologie und Rheumatologie, Charité – Universitätsmedizin Berlin, Berlin, Deutschland

**Keywords:** Ankylosierende Spondylitis, Rückenschmerz, Diagnose, Fachassistenz, Fragebogen, Ankylosing spondylitis, Back pain, Diagnosis, Nurse, Questionnaire

## Abstract

**Hintergrund:**

Der oft langsame Beginn einer axialen Spondyloarthritis (axSpA), die initial zum Teil wenig spezifischen Symptome (Rückenschmerzen), aber auch begrenzte Ressourcen und die damit verbundenen Verzögerungen in der rheumatologischen Versorgung sind Faktoren, die zu verspäteter Diagnose und Therapie dieser meist jungen Patienten mit beitragen. Rheumatologische Fachassistenten (RFA) können zur Verbesserung der Versorgung beitragen, indem sie vom Rheumatologen delegierte ärztliche Leistungen übernehmen.

**Ziel der Arbeit:**

Ziel ist, zu untersuchen, ob geschulte RFA bei Patienten mit chronischem Rückenschmerz und noch unklarer Diagnose mithilfe eines strukturierten Fragebogens anamnestische und klinische Befunde wie Rheumatologen erheben können.

**Material und Methoden:**

In der multizentrisch durchgeführten PredAS-Studie wurden bei Patienten mit dem Leitsymptom chronischer Rückenschmerz demografische Basisdaten, Anamnese und patientenberichtete Endpunkte mittels strukturierter Fragebögen von RFA und Rheumatologen unabhängig voneinander erfasst. Zudem wurden Funktion (BASFI) und Wirbelsäulenbeweglichkeit (BASMI) standardisiert gemessen. Um die mögliche Erleichterung durch Nutzung digitaler Medien zu testen, wurden 2 Patientengruppen getrennt untersucht: Die Ergebnisse der einen Kohorte wurden mittels papierbasierter Case Report Forms (CRF) und die Ergebnisse der anderen elektronisch mittels iPad dokumentiert. Die Konkordanz der Dokumentationen zwischen RFA und Rheumatologen wurde als Kappa-Koeffizient, als prozentuale Übereinstimmung und auf individueller Patientenebene berechnet.

**Ergebnisse:**

Bei fast drei Viertel der 141 Patienten mit chronischen Rückenschmerzen wurden Charakteristika des entzündlichen Rückenschmerzes identifiziert. Die Konkordanz bei Dokumentation durch RFA und Arzt war bei den anamnestischen Angaben zum Rückenschmerz höher als bei der Angabe zur Lokalisation des Rückenschmerzes. Bei der Erhebung des BASMI zeigte sich kein Unterschied zwischen RFA und Arzt (ICC 0,925) (95 %-CI 0,879–0,953). Der Zeitaufwand für die strukturierte Dokumentation betrug beim Arzt 20 ± 6,7 min und bei der RFA 28,5 ± 13 min.

**Diskussion:**

Die Ergebnisse sprechen dafür, dass geschulte RFA die Rheumatologen bei der anamnestischen Aufarbeitung und ersten körperlichen Untersuchung im Rahmen der Diagnosestellung erheblich und qualifiziert unterstützen können.

## Abstract (en)

**Background:**

The often slow onset of ankylosing spondylitis (AS), the initially partially unspecific symptoms (back pain) and the scarcity of resources in rheumatological care are important factors leading to delayed diagnosis and treatment of these mostly young patients in Germany. Qualified nurses specialized in rheumatology might improve quality of care by providing medical services delegated by the rheumatologists.

**Objective:**

The aim was to investigate whether qualified nurses specialized in rheumatology can interpret anamnestic and clinical findings such as rheumatologists in patients with chronic low back pain and still unclear diagnosis using a structured questionnaire.

**Material and methods:**

In the multicenter PredAS study a structured anamnestic questionnaire was applied independently by qualified nurses specialized in rheumatology and rheumatologists to patients referred to rheumatology practices with the leading symptom of low back pain. The questionnaire covered basic demographic data, medical history and patient reported outcomes. Additionally, measurements of physical function using the Bath ankylosing spondylitis functional index (BASFI) and spinal mobility using the Bath ankylosing spondylitis metrology index (BASMI) were standardized. In order to test the possible facilitation by using digital media, the results of two patient groups were separately documented on paper-based report forms and on an i‑pad. Concordance between documentation by qualified nurses specialized in rheumatology and rheumatologists was studied by calculating Cohen’s kappa, intraclass correlation coefficients (ICC) and percentage agreement on an individual patient level.

**Results:**

Nearly 75% of the 141 patients with chronic low back pain were identified as having the characteristics of inflammatory back pain. The concordance of the documentation for the anamnesis of back pain by qualified nurses specialized in rheumatology and physicians was higher than for the localization of the back pain. The results for the BASMI showed no differences between qualified nurses specialized in rheumatology and physicians (ICC 0.925, 95 % confidence interval, CI 0.879–0.953). The time taken for the structured documentation was 20 ± 6.7 min for physicians and 28.5 ± 13 min for qualified nurses specialized in rheumatology.

**Conclusion:**

The results indicate that well-trained qualified nurses specialized in rheumatology have a high potential to take over some of the workload from rheumatologists during documentation of the anamnesis and the initial physical examination in the diagnosis of ankylosing spondylitis.

## Hintergrund und Fragestellung

Die rheumatologische Versorgung in Deutschland ist unter anderem auch durch limitierte personelle Ressourcen in der Rheumatologie beeinflusst. Dies führt zu einer verspäteten Diagnosestellung und dem zu späten Beginn einer adäquaten Behandlung rheumatischer Erkrankungen [1]. Dies trifft insbesondere auf Patienten mit axialer Spondyloarthritis (axSpA) – das schließt die ankylosierende Spondylitis (AS) mit ein – zu, da chronische Rückenschmerzen als Schlüsselsymptom häufig sind und neben einer hohen klinischen Variabilität auch eine breite differenzialdiagnostische Fächerung aufweisen. Qualifiziertes Assistenzpersonal kann helfen, Ressourcen in der Rheumatologie sicherer und effizienter zu nutzen [2]. Das Aufgabenspektrum der rheumatologischen Fachassistenz (RFA) kann dabei von der strukturierten Befragung des Patienten bis hin zur körperlichen Untersuchung wie in den Niederlanden und zum Fallmanagement wie in England reichen [3–5]. Allerdings gibt es bisher wenig Evidenz dafür, dass die Erhebung der Symptome, Begleiterkrankungen, Familienanamnese sowie Erfassung von patientenberichteten Endpunkten mittels eines strukturierten Fragebogens und Erhebung der Wirbelsäulenbeweglichkeit durch eine RFA mit der ärztlichen Befunderhebung qualitativ vergleichbar sind.

In der hier vorliegenden PredAS-Studie wurde geprüft, ob geschulte RFA mithilfe eines strukturierten Fragebogens bei Patienten mit chronischen Rückenschmerzen anamnestische und klinische Befunde erheben können, die sich von denen der behandelnden Ärzte nicht wesentlich unterscheiden.

## Studiendesign und Untersuchungsmethoden

Die prospektive, multizentrische Querschnittuntersuchung wurde zwischen August 2010 und Januar 2012 in rheumatologischen Fachkliniken und Schwerpunktpraxen in Deutschland durchgeführt. In die Studie eingeschlossen wurden volljährige Patienten mit seit mehr als 3 Monaten bestehenden chronischen Rückenschmerzen unklarer Diagnose. Ausgeschlossen waren Patienten, bei denen bereits eine endgültige Diagnose in Bezug auf die chronischen Rückenschmerzen vorlag. Die Studie wurde von der Ethik-Kommission der Ärztekammer Westfalen-Lippe und der Medizinischen Fakultät der Westfälischen Wilhelms-Universität Münster zustimmend bewertet.

Im Rahmen der Studie wurden von RFA und Arzt unabhängig voneinander demografische Basisdaten, Anamnese, klinische Symptome, Familienanamnese und Begleiterkrankungen mithilfe eines strukturierten Fragebogens erfasst. Zudem wurde die Wirbelsäulenbeweglichkeit (Bath Ankylosing Spondylitis Metrology Index [BASMI]) gemessen und der im Rahmen der Routineversorgung bestimmte CRP-Wert dokumentiert [6]. Die patientenberichteten Endpunkte Bath Ankylosing Spondylitis Disease Activity Index (BASDAI), globales Patientenurteil (BAS-G) und Patient Acceptable Symptom State (PASS) wurden mittels standardisierter Fragebögen erfasst [7–9].

Um die mögliche Erleichterung durch Nutzung digitaler Medien zu testen, wurden 2 Patientengruppen getrennt untersucht: Die Ergebnisse der einen Kohorte wurden mittels papierbasierter Case Report Forms (CRF) und die Ergebnisse der anderen Kohorte wurden elektronisch mittels iPad dokumentiert. Die Zeitdauer des Patientenkontaktes wurde sowohl beim Arzt als auch bei der RFA gemessen.

Als primärer Endpunkt wurde der Unterschied der Dokumentation des BASMI bei Arzt und RFA unabhängig von der Art der Dokumentation (Papier- oder iPad-CRF) analysiert. Vor Studienbeginn erhielten die RFAs zu allen zu erhebenden Parametern eine standardisierte Schulung von insgesamt 4 h durch einen Rheumatologen.

### Statistische Methoden

Für alle dichotomen oder kategorialen Variablen wurden die prozentuale Übereinstimmung sowie der Kappa-Koeffizient (κ) ermittelt. Für alle metrischen Daten wurde die Intraklassenkorrelation (ICC) berechnet. Für die Berechnung der prozentualen Übereinstimmung aus Kontingenztafeln wurde die ärztliche Dokumentation als Goldstandard angenommen. Die Berechnung der Anzahl von abweichenden Ergebnissen zwischen Arzt und RFA erfolgte auf individueller Patientenebene.

## Ergebnisse

### Patientencharakteristika

Es wurden insgesamt 141 Patienten mit chronischen Rückenschmerzen an insgesamt 24 Zentren eingeschlossen (88 Patienten mit Papierdokumentation und 53 Patienten mit iPad-Dokumentation). Die 24 Zentren deckten die Breite der rheumatologischen Versorgung in Deutschland ab, da insgesamt 3 Kliniken mit einer bettenführenden internistischen Rheumatologie (davon eine Klinik als Universitätsklinik), 3 Kliniken mit einer bettenführenden orthopädischen Rheumatologie, 3 Gemeinschaftspraxen (davon eine als orthopädische Praxis) und insgesamt 15 Schwerpunktpraxen für Rheumatologie Patienten rekrutierten. Die demografischen und klinischen Parameter der Kohorte finden sich in Tab. 1. Etwa die Hälfte der Patienten war männlich, in einem Alter von 42,2 ± 15,0 Jahren, und bei 60,3 % lag ein erhöhter CRP-Wert vor (Tab. 1). Die chronischen Rückenschmerzen bestanden im Mittel seit 102,7 ± 136,3 (Arzterhebung) bzw. 96,6 ± 109,1 (RFA-Erhebung) Monaten. Bei 83,0 % (Arzterhebung) bzw. 80,9 % (RFA-Erhebung) hatten die Rückenschmerzen vor dem 45. Lebensjahr begonnen (Tab. 1). Bei fast drei Viertel der Patienten wurden Charakteristika des entzündlichen Rückenschmerzes identifiziert. Hinsichtlich der Einzelcharakteristika des entzündlichen Rückenschmerzes wurden am häufigsten „Besserung durch Bewegung“ und „gutes Ansprechen auf NSAR“ (jeweils 75 %) angegeben (Tab. 2).

**Table 1:** 

Variablen	Arzt (*n* = 141)	RFA (*n* = 141)	*p*-Wert
*Demografische und anamnestische Parameter*			
Geschlecht männlich, *n* (%)	70 (49,6)	72 (51,1)	0,906^b^
Alter [Jahre] (MW ± SD)	42,2 (15,0)	42,2 (15,0)	0,997^c^
BMI (MW ± SD)	25,9 (5,21)	26,3 (6,06)	0,693^c^
*Extraspinale und extraartikuläre Manifestation*			
Periphere Symptome an Gelenken und/oder Sehnenansätzen *n *(%)	68 (48,2)	79 (56,0)	0,917^b^
Arthritis *n *(%)	48 (34,0)	52 (36,9)	0,619^b^
Enthesitis *n *(%)^a^	12 (8,5) (*n* = 88)	16 (11,3) (*n* = 88)	0,410^b^
Psoriasis *n *(%)	12 (8,5)	13 (9,2)	0,834^b^
CED *n *(%)	5 (3,5)	6 (4,3)	0,122^b^
Uveitis *n *(%)	13 (9,2)	15 (10,5)	1,000^b^
*Assessment*			
BASDAI, 0–10, (MW ± SD)	5,86 (7,05)	5,71 (6,42)	0,675^c^
BAS‑G, 0–10, (MW ± SD)	7,62 (4,37)	7,60 (4,29)	0,982^c^
PASS, ja/nein	20 (14,2)	20 (14,2)	0,985^b^
*Labor*			
HLA-B27 positiv [*n*] (%)	61,2 (60) (*n* = 98)	60 (60) (*n* = 100)	0,771^b^
CRP Normbereich, ja/nein, *n* (%)	56 (39,7)	50 (35,5)	0,461^b^
*Familienanamnese*			
AS bei Verwandten *n* (%)	24 (17,0)	22 (15,6)	0,747^b^
Psoriasis bei Verwandten *n* (%)	13 (9,2)	19 (13,5)	0,260^b^
CED bei Verwandten *n* (%)	136 (96,5)	122 (86,5)	0,122^b^
Uveitis bei Verwandten *n* (%)	4 (2,8)	4 (2,8)	1,000^b^

*AS* ankylosierende Spondylitis, *CED* chronisch entzündliche Darmerkrankung, *na* nicht zutreffend, *SD* Standardabweichung

Einheiten, sofern nicht anders angegeben, in *n* (%)

^a^Enthesitis wurde nur im Papier-CRF abgefragt

^b^Chi-Quadrat-Test ohne fehlende Angaben

^c^Wilcoxon-Test für verbundene Stichproben ohne fehlende Angaben

**Table 2:** 

Variablen	Arzt (*n* = 141)	RFA (*n* = 141)	Kappa	Prozentuale Übereinstimmung (%)
*Charakteristika des Rückenschmerzes*				
Dauer des Rückenschmerz [Monate] (MW ± SD)	102,7 (136,3)	96,6 (109,1)	–	–
Prädominant axial *n *(%)	119 (84,4)	(109 77,3)	0,566	89,2
Alter <45 Jahre bei Schmerzbeginn, *n *(%)	117 (83,0)	114(80,9)	0,923	97,8
Langsamer Schmerzbeginn *n *(%)	100 (70,9)	97 (68,8)	0,599	82,0
Morgensteifigkeit vorhanden *n *(%)	94 (66,7)	103 (73,0)	0,637	84,2
Schmerzbesserung durch Bewegung *n *(%)	100 (70,9)	101 (71,6)	0,643	84,9
Keine Schmerzbesserung durch Ruhe *n *(%)	65 (46,1)	83 (58,9)	0,382	64,7
Nächtliches schmerzbedingtes Erwachen *n *(%)	95 (67,4)	98 (69,5)	0,837	92,8
Wechselnder Gesäßschmerz *n *(%)	80 (56,7)	83 (58,9)	0,627	81,2
Schmerzbesserung unter NSAR *n *(%)	101 (71,6)	101 (71,6)	0,768	89,9
*Lokalisation des Schmerzes*				
HWS	45(31,9)	51(36,2)	0,791	89,9
BWS	43(30,5)	53(37,6)	0,795	90,0
LWS	120(85,1)	119(84,4)	0,901	98,4
Becken	89(63,1)	85(60,3)	0,607	82,3

*BWS* Brustwirbelsäule, *HWS* Halswirbelsäule, *LWS* Lendenwirbelsäule, *NSAR* nichtsteroidale Antirheumatika

Bei 68 (Arzterhebung) bzw. 79 Patienten (RFA-Erhebung) bestand anamnestisch bereits eine extraspinale Manifestation (Arthritis bei 48 [Arzterhebung] bzw. bei 52 Patienten [RFA-Erhebung] und bei 30 [Arzterhebung] bzw. 34 Patienten [RFA-Erhebung] eine extraartikuläre Manifestation wie chronisch entzündliche Darmerkrankung [CED] 5 [Arzterhebung] bzw. 6 Patienten [RFA-Erhebung], Psoriasis 12 [Arzterhebung] bzw. 13 Patienten [RFA-Erhebung]) oder Uveitis 13 (Arzterhebung) bzw. 15 Patienten (RFA-Erhebung) (Tab. 1). Bei bis zu 17 % der Patienten lag eine positive Familienanamnese für Erkrankungen aus dem Formenkreis der SpA vor: 24 (Arzterhebung) bzw. 22 Patienten (RFA-Erhebung), im Detail Psoriasis: 13 (Arzterhebung) bzw. 19 Patienten (RFA-Erhebung), CED: 5 (Arzterhebung) bzw. 11 Patienten (RFA-Erhebung) und Uveitis: 4 (Arzterhebung) bzw. 4 Patienten (RFA-Erhebung).

### Assessment

Bei insgesamt 85 Patienten (60,3 %) lagen Fragebögen zur Auswertung vor (fehlende Werte von 25 % im Papier-CRF und 60 % im iPad). Bei den Patienten lagen eine hohe Krankheitsaktivität (gemessen mit dem BASDAI) und eine eingeschränkte Globalbeurteilung vor (Tab. 1).

### Messung der Wirbelsäulenbeweglichkeit

Bei 73 Patienten (Arzterhebung) bzw. 83 Patienten (RFA-Erhebung) lagen Angaben zur Wirbelsäulenbeweglichkeit vor (fehlende Werte von 33 % im Papier-CRF und 73 % im iPad). Bei den Patienten lag eine mäßige Einschränkung der Wirbelsäulenbeweglichkeit vor (Tab. 3): BASMI 3,0 ± 1,84 (Arzterhebung) bzw. 3,1 ± 1,98 (RFA-Erhebung).

**Table 3:** 

Wirbelsäulenbeweglichkeit	Arzt	RFA	ICC (95 %-CI)
BASMI, 0–10, (MW ± SD)	3,00 (1,84)	3,12 (1,98)	0,925 (0,879–0,953)
Anteriore Flexion, MW ± SD	6,81 (7,56)	6,98 (8,41)	0,758 (0,673–0,823)
Laterale Flexion rechts, (MW ± SD)	19,1 (14,2)	18,0 (12,5)	0,758 (0,673–0,823)^a^
Laterale Flexion links, (MW ± SD)	19,3 (14,2)	17,8 (12,8)	0,758 (0,673–0,823)^a^
Hinterkopf-Wand-Abstand, (MW ± SD)	3,35 (5,52)	3,52 (5,62)	0,908 (0,873–0,934)
Zervikale Rotation rechts [Grad], (MW ± SD)	69,8 (16,8)	70,0 (17,8)	0,931 (0,904–0,950)^a^
Zervikale Rotation links [Grad], (MW ± SD)	69,2 (17,4)	69,1 (20,2)	0,931 (0,904–0,950)^a^
Thoraxexkursion, (MW ± SD)	4,44 (1,72)	4,98 (7,49)	0,880 (0,834–0,914)

*CI* Konfidenzintervall, *ICC* Intraclass-Korrelationskoeffizient, *MW* Mittelwert, *RFA* rheumatologische Fachassistenz, *SD* Standardabweichung

Werte, sofern nicht angegeben, in cm

^a^In der Dokumentation wurde der Parameter für rechts und links getrennt erfasst. Für die Berechnung des ICC wurde der Mittelwert aus rechts und links berechnet

### Übereinstimmung der Erhebungen zwischen Arzt und rheumatologischer Fachassistenz (RFA)

Es zeigte sich im primären Endpunkt kein Unterschied zwischen der Dokumentation der RFA und des Arztes in Bezug auf den BASMI (*p* = 0,203). In den anderen untersuchten Parametern zeigte sich ebenfalls eine überwiegend sehr gute Konkordanz der Erhebungen zwischen RFA und Arzt (Tab. 3). Die Konkordanz war bei den anamnestischen Angaben zum Rückenschmerz höher als bei der Angabe zur Lokalisation des Rückenschmerzes (Tab. 2). Die Übereinstimmung der Werte der Messung der Wirbelsäulenbeweglichkeit war mit Ausnahme der lumbalen Flexion exzellent (ICC-Werte ≥0,8). Die absolute Übereinstimmung war beim Hinterkopf-Wand-Abstand (96 %) numerisch höher als bei der lateralen Flexion (35 %).

Zusätzlich zur Konkordanzanalyse einzelner Items auf Gruppenniveau wurden die Erhebungen von RFA und Arzt auf individueller Ebene getrennt berechnet. Diese interindividuellen Diskrepanzen waren bei fast allen Erhebungen in vergleichbar geringem Maß vorhanden (Tab. 3).

### Zeitaufwand der Erhebung zwischen Arzt und RFA

Der Zeitaufwand für die strukturierte Dokumentation betrug beim Arzt 20 ± 6,7 min und bei der RFA 28,5 ± 13 min. Signifikante Unterschiede zwischen iPad- und Papierdokumentation bestanden nicht.

## Diskussion

In der PredAS-Studie konnten Machbarkeit und Validität der strukturierten Erhebung durch RFA zur optimierten Vorbereitung der Diagnosestellung bei Patienten mit chronischen Rückenschmerzen in der rheumatologischen Versorgung nachgewiesen werden. In dieser Studie unterschied sich die Dokumentation anamnestischer Angaben durch die RFA mittels standardisierter Fragebögen sowie Untersuchung der Wirbelsäulenbeweglichkeit nicht wesentlich von der Dokumentation des Arztes. In der PredAS-Studie lieferten Kappa-Koeffizienten, prozentuale Übereinstimmung und Berechnungen auf der individuellen Patientenebene ein weitgehend konsistentes Bild. In der PredAS-Studie lag die Differenz des BASMI mit einer Differenz von 0,12 weit unterhalb eines systematischen Unterschieds in der Erfassung des BASMIs zwischen Arzt und RFA. Die Unterschiede im BASMI-Summenscore, die durch die Erhebung von 2 Untersuchern entstehen können („inter-reader agreement“), werden beim BASMI bei Werten <1,0 als vernachlässigbar angesehen, d. h. im Rahmen eines Messfehlers interpretiert [10].

In der Literatur wird der Aspekt der Aufarbeitung anamnestischer und klinischer Angaben nur selten als mögliche Domäne der Tätigkeit einer RFA beschrieben. Der Schwerpunkt liegt auf Organisation, Edukation, Monitoring und je nach lokalen Gegebenheiten auch Intervention [11]. Dahingehend wurde insbesondere bei Patienten mit rheumatoider Arthritis solide Evidenz für eine Nicht-Unterlegenheit des Routinemonitorings durch RFA in verschiedenen europäischen Staaten und den USA berichtet [1, 3, 12]. Für AS-Patienten stehen entsprechende Untersuchungen noch aus.

Eine Schwäche der hier vorliegenden Erhebung ist, dass aufgrund des fehlenden Follow-ups keine Expertendiagnose erhoben wurde und somit der Anteil der Patienten mit AS bzw. axSpA in der Kohorte unsicher bleibt. Das primäre Studienziel fokussierte aber auch auf die Übereinstimmung der Erhebung von Patientencharakteristika in der täglichen Praxis und weniger auf die konkrete Diagnosestellung. Es war nicht das Ziel der Studie zu überprüfen, ob die RFA eine rheumatologische Diagnose stellen kann und ob diese mit der des Arztes übereinstimmt. Ärztliche Leistungen sollen ja auch nicht ersetzt, sondern nur partiell delegiert werden.

Eine weitere Schwäche der Erhebung ist die relativ hohe Anzahl fehlender Angaben für die Berechnung der Fragebögen. Die im Fragebogen als Optionalbereich gekennzeichnete Erhebung sollte wie in dem jeweiligen rheumatologischen Zentrum sonst auch üblich erhoben werden. Daher rührt die Diskrepanz zwischen Zentren mit mehr oder weniger kompletten Datensätzen und Datensätzen mit großen Lücken in den Fragebögen.

Methodisch muss man sich aufgrund der fehlenden Verblindung fragen, inwieweit alle dokumentierten Beobachtungen tatsächlich unabhängig erfolgen konnten. Auf der anderen Seite besteht die Stärke des unverblindeten Vorgehens in der guten Abbildung der rheumatologischen Routine unter Alltagsbedingungen. Grundsätzlich ist eine Verblindung in der rheumatologischen Routine weder realistisch noch wünschenswert, da gerade die enge Zusammenarbeit von Arzt und RFA dazu dient, Abläufe zu optimieren und die Versorgungsqualität zu sichern.

Gleichwohl sind die Ergebnisse diese Studie innerhalb der aktuellen Versorgungssituation nicht 1:1 übertragbar, da ärztliche Tätigkeiten nicht automatisch an nichtärztliches Fachpersonal delegiert werden können und zudem die durch die RFA erbrachte Leistung nicht in einem Leistungskatalog abgebildet ist. Daher müssen haftungsrechtliche Fragen geklärt werden, bevor das Umsetzungspotenzial unserer Studie weiter angegangen werden kann. Die Einbindung der RFA in visitenvorbereitende Untersuchungen erfordert eine Änderung der Praxisorganisation, und die Veränderungen im Praxisablauf bedürfen auch einer wirtschaftlichen Analyse. Neben wirtschaftlichen Überlegungen kann aber auch die Generierung einer höheren Arbeitszufriedenheit bei der RFA durch Übernahme von visitenvorbereitenden Untersuchungen eine Rolle spielen.

Insgesamt bestätigt die PredAS-Studie das große Potenzial von gut geschulten RFA für die Patientenversorgung. Die Ergebnisse sprechen dafür, dass RFA die Rheumatologen bei der anamnestischen Aufarbeitung im Rahmen der Diagnosestellung mit visitenvorbereitenden Untersuchungen erheblich unterstützen können – ohne dabei kritische Qualitätseinbußen hinnehmen zu müssen. Entsprechend wurden solche Leistungen auch mit in das der Bundesärztekammer kürzlich von der DGRh vorgelegte Curriculum für RFA integriert.

## Fazit für die Praxis

- Die Verwendung eines strukturierten Fragebogens für die Erfassung von Symptomen und anamnestischen Angaben sowie die Vermessung der Wirbelsäulenbeweglichkeit von Patienten mit chronischen Rückenschmerzen ist in der Praxis ein sinnvolles und gut anwendbares Werkzeug.
- Die rheumatologische Fachassistenz kann ohne Verlust an Qualität den Arzt erheblich mit visitenvorbereitenden Erhebungen bei der Untersuchung von Neuvorstellungen entlasten, wenn die Abläufe standardisiert sind.
- Auf eine ausführliche Schulung ist insbesondere bei der Untersuchung der Wirbelsäulenbeweglichkeit zu achten.
